# Residual Strength and Drying Behavior of Concrete Reinforced with Recycled Steel Fiber from Tires

**DOI:** 10.3390/ma14206111

**Published:** 2021-10-15

**Authors:** David Revuelta, Pedro Carballosa, José Luis García Calvo, Filipe Pedrosa

**Affiliations:** 1 Institute for Construction Sciences Eduardo Torroja, CSIC, Serrano Galvache 4, 28033 Madrid, Spain; carballosa@ietcc.csic.es (P.C.); jolgac@ietcc.csic.es (J.L.G.C.); filipe.pedrosa@ietcc.csic.es (F.P.); 2 Universidad Politécnica de Madrid, 28040 Madrid, Spain

**Keywords:** fiber reinforced concrete, recycled fibers, steel fibers, polypropylene fibers, flexural residual strength, drying shrinkage

## Abstract

Fiber reinforcement of concrete is an effective technique of providing ductility to concrete, increasing its flexural residual strength while reducing its potential for cracking due to drying shrinkage. There are currently a wide variety of industrial fibers on the market. Recycled steel fibers (RSF) from tires could offer a viable substitute of industrialized fibers in a more sustainable and eco-friendly way. However, mistrust exists among users, based on fear that the recycling process will reduce the performance, coupled with the difficulty of characterization of the geometry of the RSF, as a consequence of the size variability introduced by the recycling process. This work compares the behavior of RSF from tires compared with industrialized steel or polypropylene fibers, evaluating the fresh state, compressive strength, flexural residual strength, and drying behavior. The concept of Equivalent Fiber Length (EFL) is also defined to help the statistical geometrical characterization of the RSF. A microstructural analysis was carried out to evaluate the integration of the fiber in the matrix, as well as the possible presence of contaminants. The conclusion is reached that the addition of RSF has a similar effect to that of industrialized fibers on concrete’s properties when added at the same percentage.

## 1. Introduction

### 1.1. The Concept of Fiber Reinforcement

Concrete is undoubtedly the most common building material in use today. There are several reasons that explain this issue: its component materials are relatively inexpensive and easy to produce; its manufacture is comparatively simple; as this is a product delivered in a semi-fluid state, it allows for great versatility terms of shaping; and it can be applied to a large number of buildings and civil engineering infrastructures. However, its use also encounters certain drawbacks, the main one being its brittleness, i.e., its low tensile strength compared to its compressive strength, its low deformation capacity until the appearance of the first crack, along with its rapid propagation.

The issue of brittleness of the materials used in construction is as old as the activity itself. Since ancient times, building materials, e.g., clay sun baked bricks, were reinforced with fibers such as horse-hair, straw, and other vegetable fibers to try to overcome this hindrance. The brittle matrix supports, surrounds and protects the fibers, providing compressive strength, while the fibers enhance the mechanical response of the composite by bridging the cracks that appear after tensile failure and providing a sort of ductile behavior post cracking.

The concept of fiber reinforcement was developed in modern times specifically for its use with cement-based materials. Asbestos cement products were introduced in the early 1900s and became widely used following the development of several mechanized manufacturing processes, such as the Hatschek, Magnani [1], and Manville [2] processes. Asbestos fibers were employed until about the 1970s when their effect on the development of lung cancer was proven.

The development of fibers specifically intended for concrete followed a different path from asbestos. Two different periods can be distinguished. The first one, up to the 1960s, can be considered a pioneering phase, with many ideas but few actual applications. It is a period of invention rather than scientific research, characterized by a growing number of patents. The first one is usually ascribed to A. Berard in 1874 [3], in California, who proposed the addition of granular waste iron to concrete. Certain other relevant examples, already in the 20th century, are Weakley [4], Meischkle-Smith [5], and Constantinesco [6]. All of them dealt with the use of steel fibers. The latter is particularly noteworthy since the fiber reinforcement parameters he recommends are somewhat similar to those of steel fiber reinforced concrete currently employed.

The second phase of the development of fiber-reinforced concrete can be considered as the period in which ascertaining the true influence of fibers in concrete properties from a scientific perspective was discovered. This phase started in the early 1960s with the research work of Romualdi et al. [7,8] in the US and Krenchel [9] in Denmark, and was expanded upon with several studies undertaken over the next decade (Shah [10], Naaman [11], Swamy [12], among others) that boosted the development of a multitude of fibers [13] and materials [14] that continues until the present as new discoveries and applications are identified [15,16].

Steel fibers are the most used type of fibers for concrete reinforcement and crack control. They have been chosen for a wide range of concrete elements such as pavements, slabs, bridge decks, beams, tunnel linings, foundations, and walls, with volume fraction rates standing at mostly below 2%. Their advantages are that they are widely available; relatively inexpensive when compared to other fibers; they present high strength, modulus, fracture toughness, and temperature resistance; and are deformable. On the downside, they are corrosion-prone, and their manufacturing cost is high if small diameters are wanted. In non-load bearing applications, such as pavements, the fiber content is usually under 0.5% by volume [17]. A common figure, used later in this paper for concrete mix design, is the addition of 20 kg of steel fibers per m^3^ of concrete (*V_f_* = 0.26%).

Polypropylene fibers were first used in concrete in the 1980s [18,19]. These are also widely available, their cost is low, and they are highly stable in a concrete matrix, still they have low modulus, medium to low strength, poor bonding capabilities and are difficult to mix in larger volumes. Consequently, their use has found a niche that could be considered as secondary reinforcement in non-structural applications, particularly to control plastic shrinkage cracking of concrete at an early age. In such applications, the volume fraction of fibers is less than 0.2% by volume, being often close to 0.1%. In this paper, an addition of polypropylene fibers of 1.0 kg/m^3^ is considered (*V_f_* = 0.11%).

Most of the steel and polypropylene fibers used for concrete reinforcement are comprised of industrial products manufactured from raw materials. Over the last two decades, the concern for the sustainability of industrial processes has led to the undertaking of numerous research efforts looking for the replacement of industrial products by recycled fibers at low cost and with less environmental impact [20,21].

### 1.2. Recycled Steel Fibers from Tires

According to the 2019 statistics of the European Tire and Rubber Manufacturers Association (ETRMA), that accounts for 70% of the world tire industry turnover; in other words, 5.1 million tons of tires were produced throughout 2018 by its members [22]. The EU Landfill Directive [23], which came into full effect in July 2006, requires that virtually all end-of-life tires (ELTs) be recycled or re-used in some way. The adoption of this policy means that, according to ETRMA, 91% of ELTs were collected and treated for material recycling and energy recovery in 2018 [24]. In the UK alone, over 40 million used tires are treated every year. Around 2 million tons (61.75% of total ELTs treated) were treated through material recovery, where secondary materials from ELTs are used in construction, automotive, and civil engineering applications.

A typical tire consists mainly of rubber (47–48% by weight) and black carbon (22%), though an important part is comprised of steel wires and cords (15–17%) that provide stiffness and resistance to the tire [25]. The remainder are fabrics and other minor additives. Steel wires and cords can be recovered and transformed into fibers. The extraction of the fibers from the tires is carried out mainly by three methods: shredding, cryogenic, and pyrolysis processes. The first two processes, called mechanical recycling, are the most commonly used. Mechanical processes damage the cables superficially to a certain extent, but they still retain the ability to transfer stresses, due to their slenderness ratio and their irregular shape, thus favoring anchorage [26].

According to these figures, ELTs suggest themselves as the potential source of more than 300,000 tons of recycled steel fibers per year. Owing to this sizeable potential, a sizable number of research works have been performed over recent decades regarding reinforcement of concrete with steel fibers recycled from tires. First attempts were carried out by Wu et al. in the US in the 1990s [27]. Their work focused on the shrinkage performance measured on ring-type specimens, as well as the flexural load vs. deflection behavior, since those are the main enhanced properties that can be expected from the use of fibers in concrete, as has been mentioned before. These works were continued both in the US [28] and in the UK at the University of Sheffield [26,29], and during the last decade, more than 150 papers have been published on recycled steel fiber reinforced cement-based composites [20].

The existing literature shows that most of the fibers used were obtained through the shredding process. A scant number of the works have focused on the geometric characterization of the fibers, reaching the conclusion that the main geometric characteristics, diameter and length, depend on the type of tire and the extraction process [30], meaning that to obtain an effective characterization it should be necessary to perform a statistical analysis [26]. The most thorough studies in this regard are those carried out in Italy by a group of researchers from the University of Salento [31,32,33]. Usual diameter and fiber length ranges are 0.15–0.26 mm and 25–40 mm, respectively [20].

**Figure 1:**
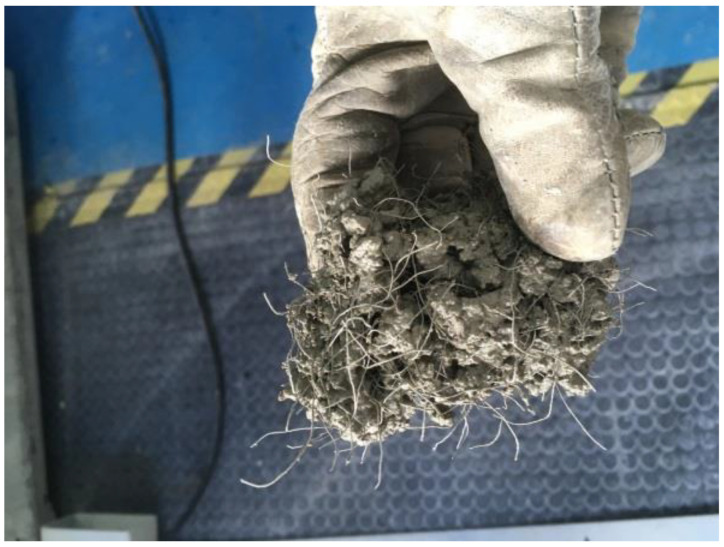
“Balling” effect of recycled steel fibers from tires (Picture by authors).

With regard to industrial steel fibers, studies show that the addition of recycled steel fibers reduces the workability of concrete mixtures, being mainly affected by the type and content of fiber [34,35]. The variety of sizes and shapes of recycled fibers makes them susceptible to the “balling” effect (Figure 1), which has caused countless issues in the applications of fibers in concrete. To avoid this problem, Aiello et al. [31] recommends to limit the volume content under 0.46%. Grunewald et al. even reduce this limit to 0.25% [36]. However, other references have used higher fiber contents without detecting any ball formation [37,38].

Regarding compressive strength, the studies show that, as with industrial steel fibers, a small addition of recycled fibers does not have a significant bearing on compressive strength [33,39], yet, after a certain threshold, increasing the fiber content decreases the compressive mechanical response [40]. This threshold would depend on the fiber and concrete types.

Finally, the state-of-the-art includes a good number of works that investigate the influence of the addition of recycled fibers on the residual strength of concrete under flexural and shear loads, once the first cracking has occurred [41,42,43,44]. Results show that recycled steel fibers provide a similar energy absorption capacity and residual strength after first cracking upon flexural loading to that of industrial fibers, when used in similar volume contents.

However, although the review of the state-of-the-art shows that it is possible to obtain a sustainable and eco-friendly fiber-reinforced concrete through the use of recycled fibers from tires, its use is not widely implemented at present in the construction sector, despite the large potential source derived from the large number of ELTs processed each year. Most of the studies point out the distrust of the users when considering a recycled product, due to the fear of a decrease in properties compared to an industrial product [21], as one of the reasons for this slow introduction. Furthermore, although most of the works have focused on the fibers’ structural behavior, especially with regard to the compression and flexural behavior of fiber-reinforced concrete, as well as aspects related to workability, other relevant properties such as durability, shrinkage, or creep require further investigation in the future, with the amount of data regarding these properties remaining limited. Interestingly, the first study on the use of recycled fibers focused on the characterization of the improving resistance to shrinkage cracking, which has hardly been studied since. Likewise, the geometric characterization of the fibers, of major significance from their application viewpoint, lacks unification when defining and measuring the main geometrical characteristics of the fibers, taking into account that the extraction process can generate great variability in these properties.

To overcome these drawbacks, the purpose of this work is to increase the knowledge on the behavior of recycled fibers from tires compared to the behavior of two widely accepted and trusted types of industrial fibers, steel and polypropylene. The first type is the most common material used for concrete reinforcement from the structural outlook, while polypropylene fibers are a common solution to protect concrete from the appearance of cracks due to shrinkage. This final aspect has hardly been considered in previous works dealing with recycled fibers. Likewise, a procedure for characterizing the two main geometric properties of the fibers, diameter and effective length, is proposed, in order to be able to proceed to their classification.

## 2. Materials and Methods

### 2.1. Materials and Concrete Mixes

**Figure 2:**
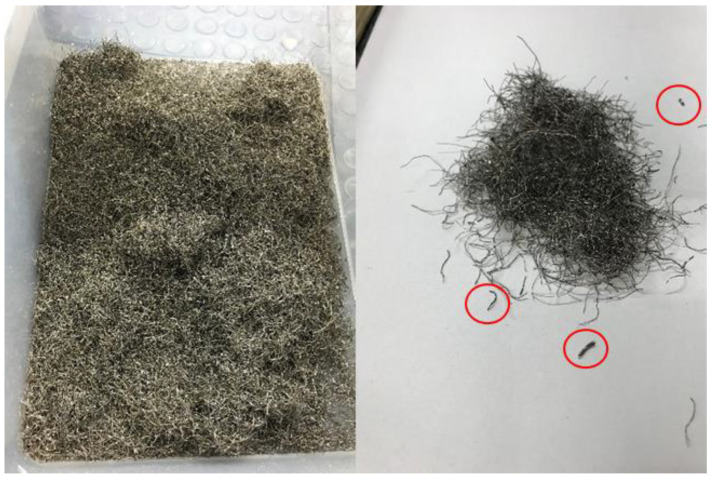
Recycled steel fibers from tires used in this paper.

**Figure 3:**
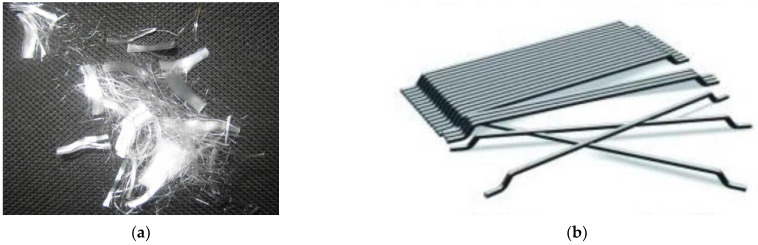
Common industrial fibers used in this paper: (**a**) polypropylene fibers; (**b**) steel fibers.

Recycled steel fibers (RSF) from tires were used in this paper for internal reinforcement of concrete. RSF were obtained by shredding and separation (Figure 2). Their geometrical characteristics are described later. In order to establish the performance of RSF with respect to those widely used in concrete, two of the most common types of fibers used in fiber-reinforced concrete were selected, such as polypropylene monofilament fibers to control cracking (Figure 3a), and steel fibers (Figure 3b) to provide residual strength after cracking of concrete. Polypropylene fibers (PPF) (Master Builders Solutions España, S.L.U., Cornellà de Llobregat, Barcelona, Spain) in line with European standard EN 14889-2 [45] and steel fibers (SF) (Industrias del Ubierna, S.A., Barcelona, Spain) according to EN 14889-1 [46] were used, their basic characteristics being displayed in Table 1.

To appraise the fibers’ behavior in concrete, four different concrete mixes were devised (Table 2). In order to determine the ability of RSF to control shrinkage cracking, two concrete mixes containing the same volume (*V_f_* = 0.11%) of RSF and PPF were used, named RSF-CC and PPF-CC. To assess the ability to provide residual flexural strength after the appearance of the first crack, two other mixes containing 20 kg/m^3^ of fiber (*V_f_* = 0.26%) were used, named RSF-RS and SF-RS.

Composition and characteristics of the mixes followed the recommendations established in the European standard EN 14845-1 [47]. Ordinary Portland cement (Portland Valderribas, Morata de Tajuña, Madrid, Spain) of the class CEM I 42,5R according to the European standard EN 197-1 [48] was used. Aggregates were siliceous 6/12 gravel (minimum/maximum particle size) and siliceous 0/4. A superplasticizer admixture (Master Builders Solutions España, S.L.U., Cornellà de Llobregat, Barcelona, Spain) as per European standard EN 934-2 [49] was incorporated in order to foster the mixes’ workability.

### 2.2. Geometrical Characterization

Geometrical properties of the fibers affect their reinforcement capacity. According to Naaman [17], a simple relationship to consider when optimizing the performance of fiber-reinforced concrete in tension, bending, shear, and compression takes the form:

(1) $$\Lambda \times \tau \times V_{f}\times L/d,$$

whereby *Λ* is a coefficient which is the product of several other coefficients (orientation, statistical distribution, group effect, snubbing, efficiency, etc.); *τ* is the bond at the matrix interface; *V_f_* is volume of fiber per unit volume of composite; and *L*/*d* is the fiber aspect ratio, that is, the length of the fiber over its diameter.

Because of the influence of the *L*/*d* parameter on the mechanical response of the composite, one of the main issues with RSF is their geometrical characterization. Due to its origin from different wires and its extraction process, the fibers come in a variety of diameters and lengths, twisted or with hooked ends. As a result of these diverse presentations, the most suitable characterization of the geometric properties of RSF seems to be a statistical description of the type of a frequency distribution or the plotting of a histogram of the diameter and length of the fibers.

Previous works [31,32,42] have characterized the diameter of the fibers by averaging two or three measurements, namely at the extremes and at the mid length of each fiber. This procedure may be suitable for very long fibers, though for short fibers (Figure 2, fibers circled in red) where the fiber length is of the order of magnitude of the equipment measurement platform (~5 mm), obtaining three measurements at three different points may not be feasible. In the opinion of this paper’s authors, deduced from what they have observed, the diameter of the fibers does not vary so much along the length of the fiber as to require calculating an average over a single fiber, since a logical geometric characterization will consist of a frequency diagram or histogram with bins of a size much larger than the variability of the diameter within the fiber. Thus, it is estimated that a single diameter measurement per fiber is sufficient to classify it within its corresponding bin, without the need to take more measurements to increase the accuracy of the characterization.

**Figure 4:**
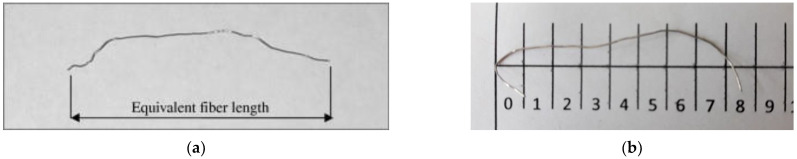
Examples of RSF length characterization: (**a**) from [32]; (**b**) used in this paper.

Regarding the fibers’ length, characterization may be even more complicated than the diameter, due to the difficulty of measuring a linear dimension on a fiber that, being bent or twisted, occupies a 3D space. The above-mentioned works, as well as some design guides [50], solved this issue by establishing an equivalent fiber length as the distance between the outer ends of the fibers. However, this definition is deemed incomplete, since the fibers characterized in [31,32,42] appear to be predominantly two-dimensional, which facilitates the measurement of the equivalent length, and they do not contain hooked ends (Figure 4a). However, the RSF used in this work often have hooked ends, and they are not clearly contained in a plane, which makes it difficult and not quite realistic to determine the equivalent length as the distance between ends (Figure 4b).

**Figure 5:**
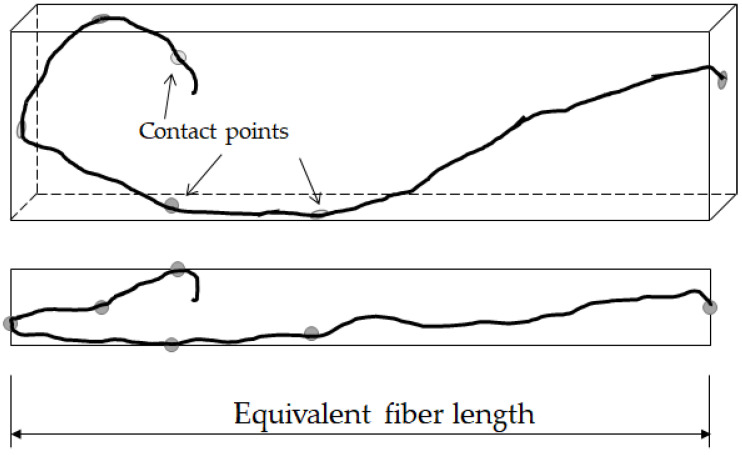
Definition of an Equivalent Fiber Length (EFL).

To overcome this drawback, the authors of this work propose a definition of the Equivalent Fiber Length (EFL) of an RSF as the length of the longest edge of the rectangular cuboid that contains the fiber (Figure 5). Although the exact EFL is still difficult to determine, the characterization of the EFLs of a batch of fibers is not so complicated from the practical standpoint. Since for the determination of the histogram it is only necessary to know the bin in which each fiber is classified, a suitable bin size should be relatively large, and the slenderness of the fibers is generally high enough, it is relatively easy to see with the naked eye the bin to which the fiber belongs by the mere projection onto a flat surface (Figure 4b).

### 2.3. Testing Methods

Characterization of the fresh state was performed by determining the consistency of each of the mixtures by the slump test according to the European standard EN 12350-2 [51], density according to EN 12350-6 [52] and the air content in line with EN 12350-7 [53].

For each of the four concrete mixes, compressive strength was determined according to European standard EN 12390-3 [54] on three cylindrical specimens of size ∅150 mm × 300 mm according to EN 12390-1 [55], made and cured during 28 days prior to testing according to EN 12390-2 [56] (RH ≥ 98%; T = 20 ± 2 °C).

**Figure 6:**
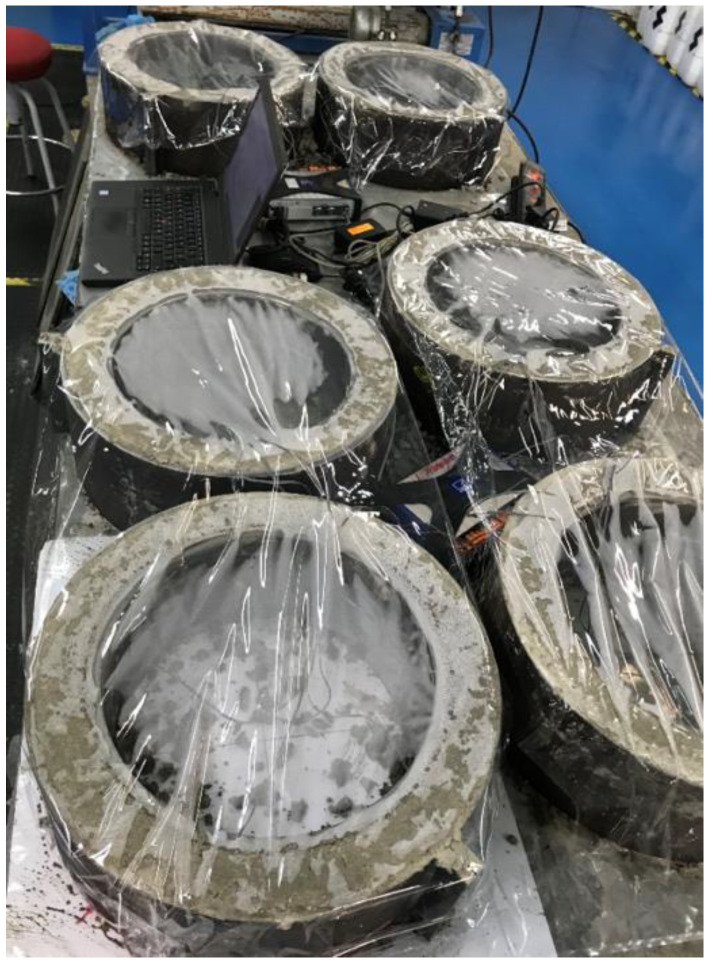
Concrete rings made to ascertain cracking resistance according to ASTM C1581 [57] (Picture by authors).

For the characterization of the cracking resistance performance of the PPF-CC and RSF-CC mixes, the standard test method ASTM C1581 [57] for determining age at cracking and induced tensile stress characteristics of concrete under restrained shrinkage was used. This method is used to evaluate the influence of the variation of the component materials and their proportions on the cracking age of the concrete as a consequence of the stresses derived from drying shrinkage and the deformations related to autogenous shrinkage and heat of hydration. Three rings were manufactured for each concrete mix (Figure 6), demolded after 24 h, and subjected to a curing environment (RH = 50 ± 5%; T = 20 ± 2 °C) after demolding. Deformations were monitored continuously on the internal steel rings by means of strain gauges, recording data every 5 min from the moment the concrete was poured and compacted by means of a vibrating needle. Monitoring was carried out over 40 days.

**Figure 7:**
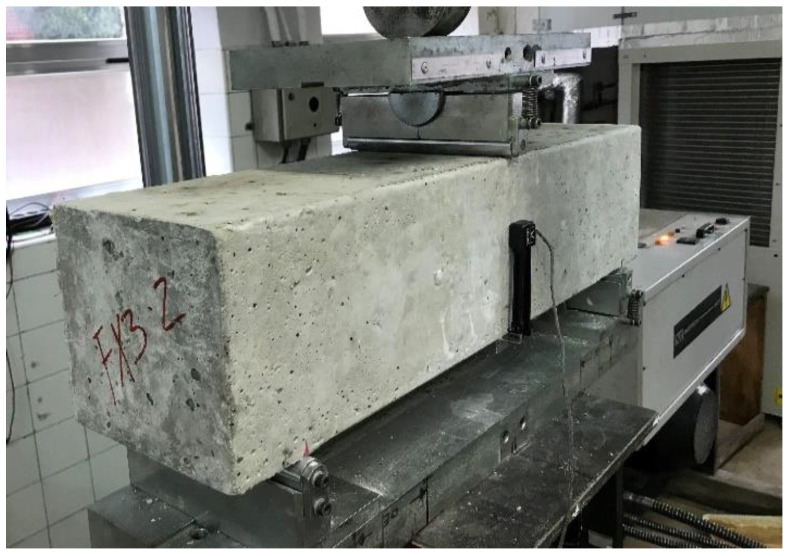
Test set up to ascertain flexural tensile strength according to EN 14651 [58] (Picture by authors).

For the characterization of the residual strength under flexural loads of the SF-RS and RSF-RS mixes, the standard test method EN 14651 [58] (Figure 7) for measuring the limit of proportionality (LOP) and the residual strength was used. For each mix, three concrete prism specimens of size 150 mm × 150 mm × 600 mm according to EN 12390-1 [55] were made following the guidelines stated in of EN 14651 [58] and cured for 28 days prior to testing according to EN 12390-2 [56] (RH ≥ 98%; T = 20 ± 2 °C). Specimens were sawed in their mid-section in order to hasten crack initiation.

The number of specimens per mix for the characterization of the compressive strength, cracking resistance, and flexural tensile strength was taken from the recommendations in the standards or in the guides for the design of fiber reinforced concrete [50,59,60].

Finally, the integration of the RSF in the concrete matrix was assessed via microscopic observation. Back-Scattered Electron Microscopy (BSEM) with Energy Dispersive X-ray Analysis (EDAX) were made in small concrete pieces taken from both RSF-CC and RSF-RS mixes. The samples, once embedded into an epoxy resin, cut, polished, and coated with carbon were inserted in a scanning electron microscope Hitachi S-4800. This equipment has an energy dispersive analyzer BRUKER 5030.

## 3. Results and Discussion

### 3.1. Geometrical Characterization

**Figure 8:**
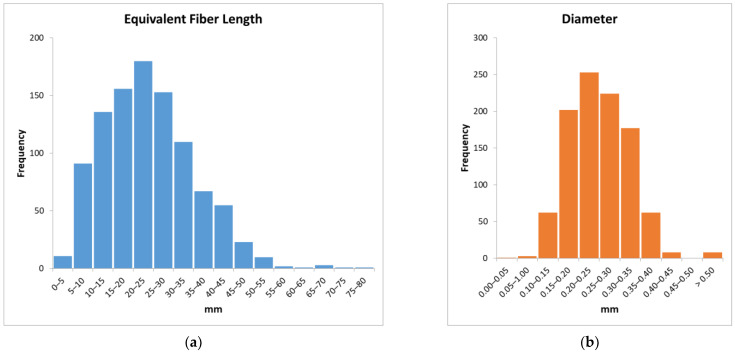
Histograms of geometrical characteristics: (**a**) Equivalent Fiber Length; (**b**) diameter.

Attending to the aforementioned procedures for geometrical characterization, a random sample of 1000 fibers was measured. The sizes of the bins were established at 0.05 mm for diameters and 5 mm for Equivalent Fiber Lengths. Figure 8 shows both histograms. Of the fibers used in this work, 90% have EFLs between 5 mm and 45 mm and diameters between 0.15 mm and 0.40 mm. The most common EFL (18.00% of the fibers) is between 20 mm and 25 mm, and the most common diameter (25.30%) between 0.20 mm and 0.25 mm. These figures are similar to the reported geometric characteristics of fibers used in previous works [31,32].

### 3.2. Fresh State

During the mixing of the components, no fiber balls were formed with any type of fiber, including RSF, which could have compromised workability.

**Figure 9:**
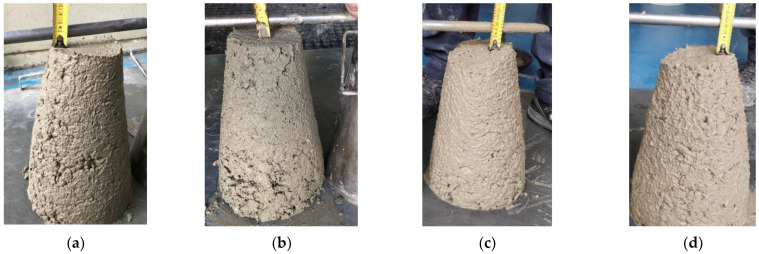
Slump-test cones from EN 12350-2 [51] test method: PPF-CC (**a**), RSF-CC (**b**), SF-RS (**c**), and RSF-RS (**d**). (Pictures by authors).

Table 3 displays a summary of the results obtained in the characterization of the fresh state. Figure 9 shows pictures of the slump-test cones. It can be concluded that the use of RSF in the same volumetric ratios selected to control cracking or to provide flexural residual strength does not lead to a significant change in the fresh state properties compared to the use of PPF or SF, since the workability class does not change from a practical standpoint, while the density and air content do not vary appreciably. The fresh state behavior is also similar between the control cracking mixes and the residual strength mixes, since there are no major changes in workability (Figure 9), density, or air content.

### 3.3. Compressive Strength

Table 4 shows the compressive strength results after 28 days of curing (RH ≥ 98%; T = 20 ± 2 °C). The values of the compressive strength determined for each of the mixes per test type are similar, without indication of a negative bearing on the compressive strength of the RSF with respect to the addition of PPF or SF. Mean and relative range are calculated as estimates of the centrality and variance of the results [61]. Within-batch variability of the three tested specimens per mix is notably below the acceptance value for most concrete codes (relative range ≤ 20%) [60]. An increase in the RSF volume content would appear to decrease compressive strength, but the number of specimens is too low as to extract any conclusions with an acceptable statistical significance.

### 3.4. Control Cracking

**Figure 10:**
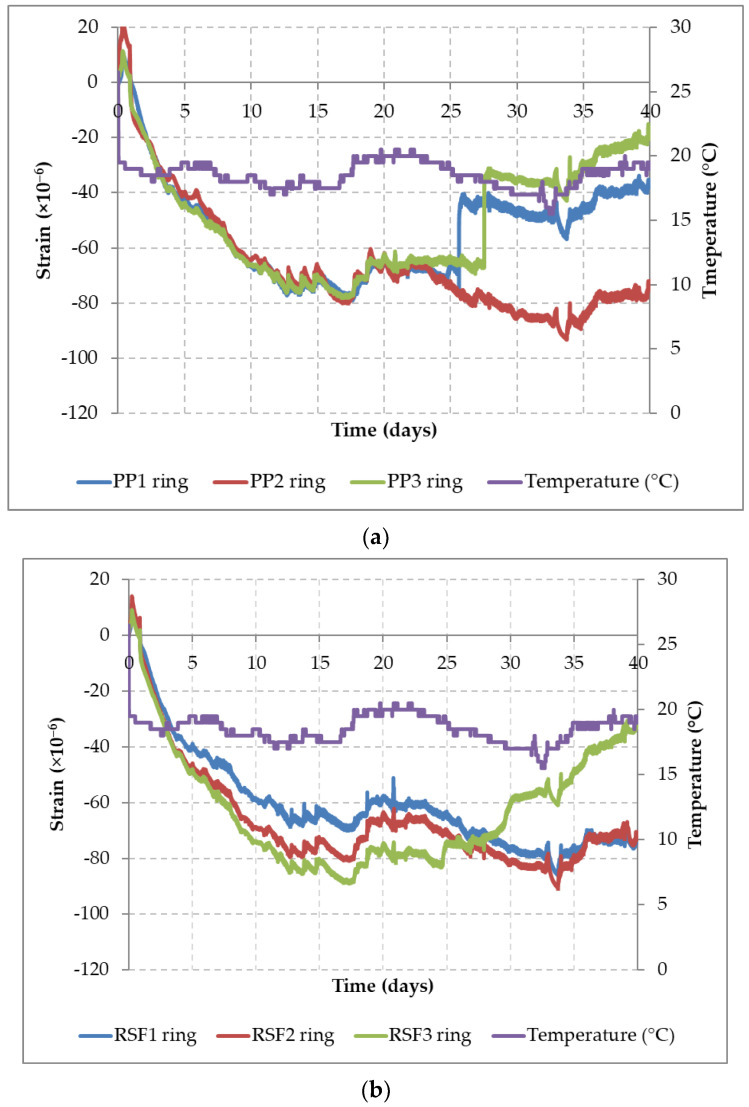
Inner ring strains recorded with the ASTM C1581 [57] test for cracking control evaluation: PPF-CC mix (**a**) and RSF-CC mix (**b**).

Figure 10 shows the restrained shrinkage curves (inner ring strains vs. time) obtained according to the ASTM C1581 [57] procedure for both concrete mixes with *V_f_* = 0.11%, PPF-CC (Figure 10a) and RSF-CC (Figure 10b). Both graphs display curves with highly similar shrinkage regimes, with a maximum negative strain around 84 μm and 89 μm. Up to the moment of the first onset of cracking, no appreciable difference in the contribution from any of the fibers, PPF or RSF, could be observed.

The initial cracking of the concrete matrix appeared at different ages, depending on the ring and concrete mix, that represented a relaxation of the strains. Once the cracking occurs, considerable differences were observed with regard to the post-cracking behavior depending on the type of fibers used. In the rings corresponding to the PPF-CC mix, it was observed that two of the three rings (PP1 ring and PP3 ring) cracked without warning at the age of 26 and 28 days, causing in practice the full decompression of the internal steel rings. On the other hand, for the rings made with the RSF-CC mix, only one of the rings gradually decompressed once the cementitious matrix cracked around day 25, without any sudden transition in the strain transfer. This post-cracking behavior indicates that the RSF provides a cracking resistance that confers ductility to the concrete. The variability in strain observed before the aforementioned ages, in which the first crack in the concrete matrix was noted, is attributed to the volume changes produced by thermal expansion caused by changes in temperature (purple line).

The ASTM C1581 [57] test method includes a classification of the potential for cracking of the concrete under study, depending on the age of first cracking after drying (*t*_cr_) and the average value of the stress development. The average stress rate (S) is the average of the stress rate for each ring specimen (*q*), calculated as:

(2) $$q=\frac{G\left|\alpha_{avg}\right|}{2\sqrt{t_{r}}},$$

whereby *G* is a constant (72.2 GPa) based on the ring dimensions used in the test method, |*α_avg_*| is the absolute value of the average strain rate factor for each ring specimen, (m/m)/day^1/2^, and *t_r_* is the elapsed time at cracking in days. The standard indicates that the concrete has a moderate-low potential for cracking if *t*_cr_ is greater than 14 days or S is less than 0.17, and a low potential for cracking if *t*_cr_ is greater than 28 days or S is less than 0.10.

Based on the classification according to ASTM C1581 and the results obtained for both mixes, PPF-CC and RSF-CC, shown in Table 5, it is established that both concrete mixes display a low potential for cracking, and that the mix containing RSF is capable of controlling early shrinkage cracking at least as effectively as PPF.

### 3.5. Residual Strength

**Figure 11:**
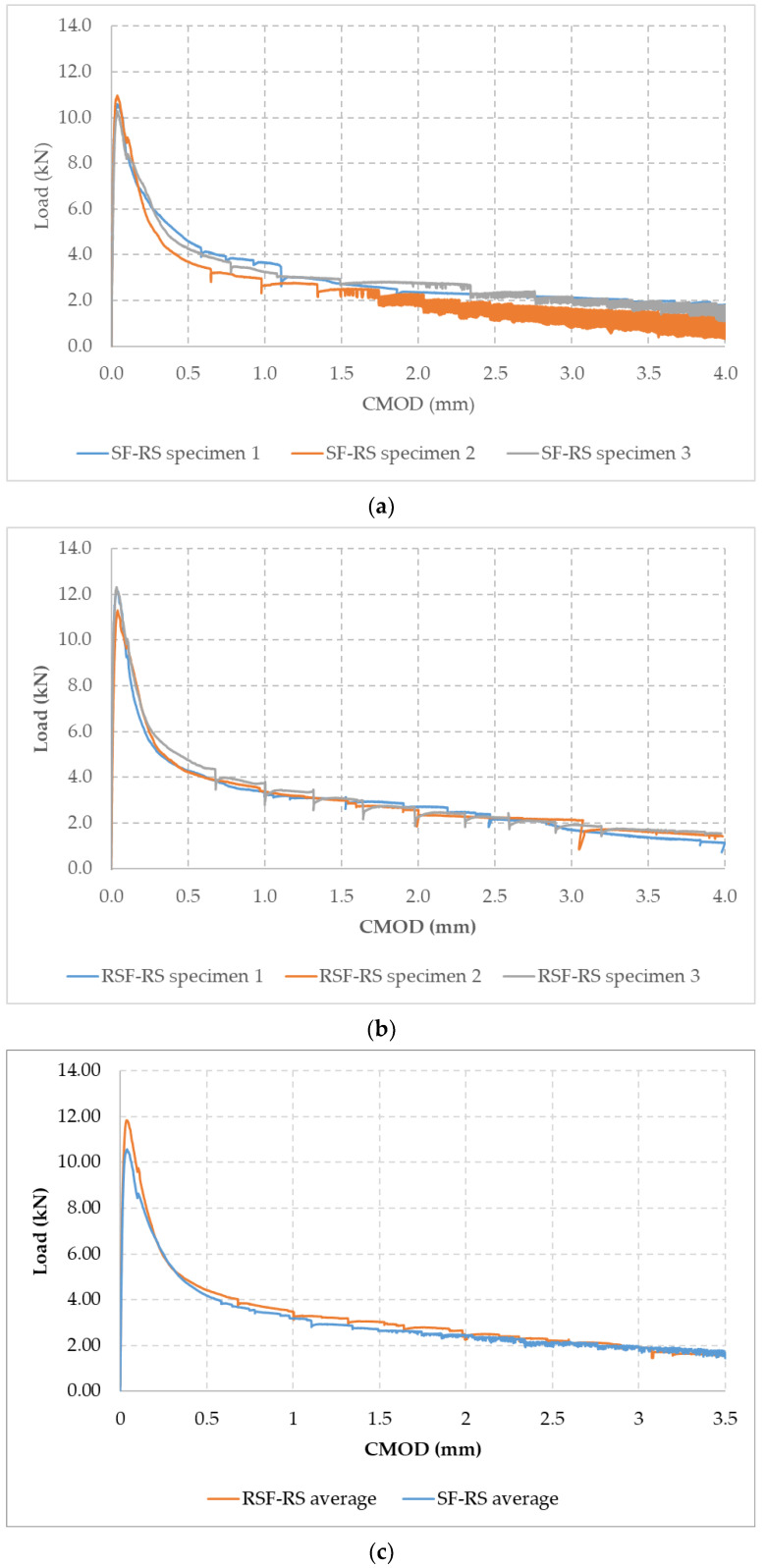
Load vs. CMOD curves according to EN 14651 [58] for determining flexural residual strength: SF-RS (**a**), RSF-RS (**b**), and average curves (mean of the three specimens per mix) for both mixes (**c**).

Figure 11 shows the load vs. Crack Mouth Opening Displacement (CMOD) curves obtained for each specimen tested according to the EN 14651 procedure [58], for both concrete mixes with *V_f_* = 0.26%, SF-RS (Figure 11a) and RSF-RS (Figure 11b), as well as the average load-CMOD curves from the three specimens per mix (Figure 11c). Table 6 shows the loads and residual strengths obtained from the curves for the limit of proportionality (LOP), CMOD = 0.5 mm (*f*_R,1_), and CMOD = 2.5 mm (*f*_R,3_). The sudden drops in the load showed in the graphs in Figure 11, particularly from CMOD = 1.5 mm, are due to the pull-out, failure of the fibers or a combination of both, a phenomenon already reported in previous works [17].

From the results displayed in the graphs, it can be concluded that both types of fibers added at *V_f_* = 0.26% (20 kg/m^3^) provide ductility to concrete after the appearance of the first crack, resulting in a deflection-softening behavior (while maintaining a constant rate of crack’s mouth opening, the load applied in bending decreases gradually following the appearance of the first crack).

**Figure 12:**
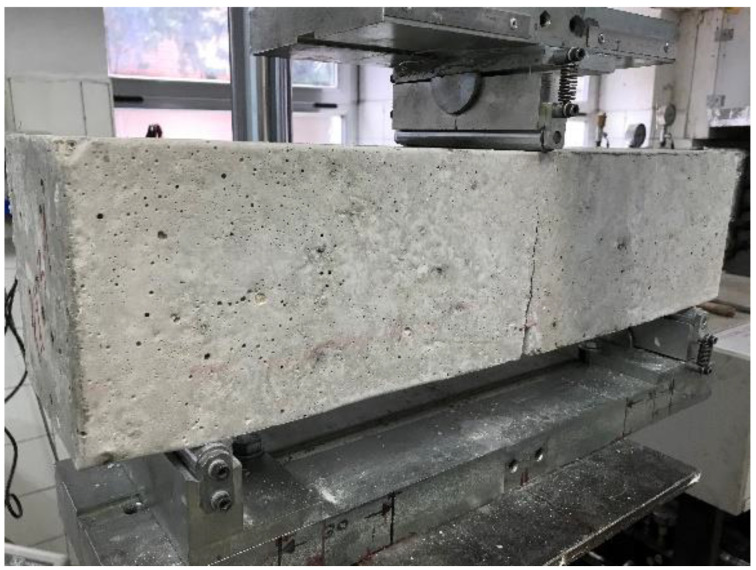
Location of the crack at the center spam in one of the tested beams (Picture by authors).

All tested prisms were accepted as valid results, since the crack appears in the center spam of the beam starting in the sawed section (Figure 12).

Dispersion of the results is, as is to be expected, higher than in the compression tests. Although the standard EN 14651 [58] does not provide precision data, the dispersion of the results can be considered low for each of the tested mix according to previous observations [17], which report that standard deviations up to 30% are normal. The curves obtained in this work for each tested prism practically overlap, while the relative ranges of the calculated LOP and residual strengths suggest a uniform distribution for both types of fibers SF and RSF. SF-RS and RSF-RS show a highly similar post-cracking behavior, meaning it can be stated that the contribution of the fibers to flexural residual strength of the concrete after first cracking is in the same order of magnitude. RSF seems to increase mechanical performance slightly, although the statistical power of the experiment is overly low due to the high dispersion of the test as to evaluate this effect with sufficient statistical confidence.

Thus, according to the results obtained for both mixes SF-RS and RSF-RS shown in Table 6, it is established that the addition of both types of fibers in a *V_f_* = 0.26%, RSF and SF, modifies the post-cracking behavior of concrete in a similar way, providing ductile behavior and residual strength after the first crack appears.

### 3.6. Microstructural Characterization

**Figure 13:**
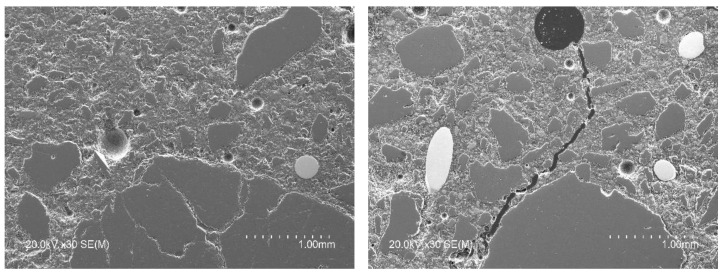
Recycled steel fiber (elements with spherical or elliptical shape in in light gray color) in the cementitious matrix.

BSEM with EDAX microanalysis was used for microstructural evaluation of the interfaces of the concrete matrix and the RSF. Figure 13 shows, in two images, the presence of RSF with a mean diameter of 270 µm (circumferential diameter measured on some samples observed in SEM) perfectly integrated in the cement matrix.

**Figure 14:**
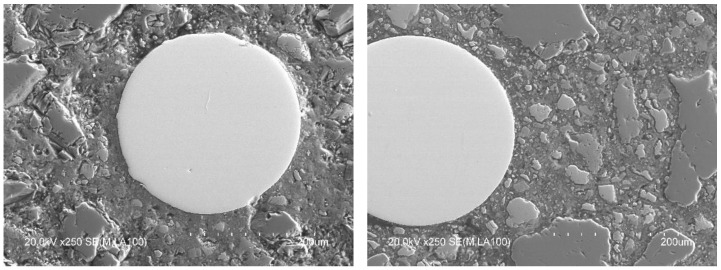
Detail of the fiber-matrix interface and appearance of the matrix component phases.

The fiber-matrix interface did not unearth any type of gap that could compromise the adherence and transmission of stress between both phases. This can be seen in detail in the images of Figure 14 where a recycled steel fiber is shown surrounded by a dense cement matrix. Moreover, the hydrates closer to the fiber feature a highly similar morphology to those of other areas of the concrete matrix away from the fiber-matrix interface.

**Figure 15:**
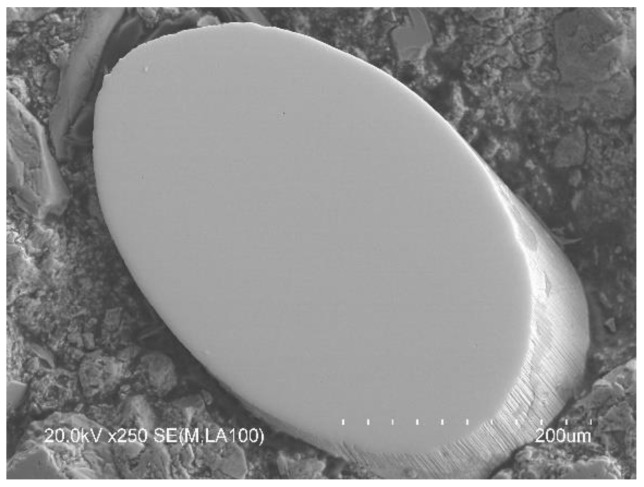
Detail of the surface of the recycled steel fiber embedded in the cementitious matrix.

Regarding the RSF, Figure 15 shows that the fibers have a ridged outer surface, an issue that contributes to the mechanical adherence between the fiber and the cement matrix.

It must be taken into account that the process of obtaining RSF from tires by shredding is a crumbling process that could introduce some level of damage to the fibers and cannot guarantee the same level of uniformity as an industrially manufactured steel fiber. Indeed, during the supply of the RSF used in this paper, during visual inspection of the received material, some rubber residue adhered to the fibers was occasionally found and a copper-like hue was observed (Figure 2), which subsequently disappeared during mixing with the rest of the components of the concrete mix.

This apparent contamination could be a potential modifier of the chemical composition of the cement paste in the interface with the RSF. For example, the presence of certain inorganic chemical elements associated with rubber manufacture (such as Sulphur S and Zinc Zn) in unusual percentages in conventional cement pastes could compromise the fiber-matrix adherence [62,63]. Thus, in order to detect differences in the chemical composition between the cement matrix and the interface area, EDAX micro-analyses were carried out.

More than 80 specific EDAX microanalyses were performed on the cementitious matrix and on a ring that surrounds the interface with the fiber. The microanalyses were carried out from the fiber up to approximately 30 µm away. No appreciable differences were detected between the chemical composition of the cement paste and the RSF-matrix interface, with highly similar Al/C and S/C ratios. Only in a few of the areas analyzed, a slight increase in the C/Si ratio in the ring near the RSF-matrix interface was detected. In turn, unconventional presence of elements such as Zn and S in the matrix or in the interface was not detected.

Thus, according to the BSEM images and EDAX microanalysis taken on the fiber-reinforced concrete samples studied, good integration and adhesion of the RSF in the cementitious matrix is deduced. This good integration of the RSF, despite its intricate shape, and bonding with the cementitious matrix, is then corroborated by the results obtained in the residual tensile strength tests carried out according to the standard test method EN 14651 [58].

## 4. Conclusions

Based on the results showed in this paper, the following remarks can be drawn:

1. The use of recycled steel fibers (RSF) from tires is viable, thus obtaining a sustainable and eco-friendly concrete.
2. The variability in the shape and length of the RSF obtained by a shredding process makes a statistical analysis necessary to characterize their geometric properties. To aid in this characterization, the concept of Equivalent Fiber Length (EFL) has been defined as the length of the longest edge of the rectangular cuboid that contains the fiber.
3. The addition of RSF has a similar effect on the workability of concrete as the addition of industrial fibers such as steel (SF) and polypropylene fibers (PPF), when added in similar proportions.
4. RSF have the same effect on compressive strength as industrial fibers SF and PPF, when added in the same proportions. As with industrial fibers, there appears to be a fiber content threshold beyond which a decrease in strength occurs.
5. The addition of RSF modified the brittle behavior of plain concrete under flexural loads, providing ductility to the material. RSF offer similar performance as industrial SF for low contents by volume (*V_f_* = 0.26%), providing a residual strength once the first crack appears in the concrete.
6. RSF can control drying shrinkage cracking during the first days as concrete in a similar way as industrial PPF for the same content of fiber in terms of volume, thereby reducing the potential for cracking.
7. Microstructural analysis has shown a good integration and adhesion of the RSF with the cementitious matrix, without the presence of potential contaminants.

## Figures and Tables

**Table 1:** Basic characteristics of the industrial fibers used in this paper.

Material	Density (kg/dm^3^)	Length (mm)	Diameter (mm)	Slenderness (*L*/*d*)
Polypropylene (PPF)	0.91	12	0.031	343–387
Steel (SF)	7.8	36	0.55	65

**Table 2:** Concrete mixes used in this paper.

Constituent	Content (kg/m^3^)			
	Control Cracking Tests (*V_f_* = 0.11%)		Residual Strength Tests (*V_f_* = 0.26%)	
	PPF−CC	RSF−CC	SF−RS	RSF−RS
Water	192.5	192.5	192.5	192.5
Cement	350.0	350.0	350.0	350.0
Coarse Aggregate	985.5	985.5	983.4	983.4
Sand	791.2	791.2	789.5	789.5
Superplasticizer	1.8	1.8	1.8	1.8
RSF	−	8.6	−	20.0
PPF	1.0	−	−	−
SF	−	−	20.0	−
Water/Cement Ratio (*w/c*)	0.55	0.55	0.55	0.55

**Table 3:** Fresh state properties of the concrete mixes.

Property	Control Cracking Tests		Residual Strength Tests	
	PPF-CC	RSF-CC	SF-RS	RSF-RS
Slump (mm)	10	20	25	35
Density (kg/m^3^)	2344	2351	2358	2363
Air Content (%)	2.3	2.0	2.3	2.1

**Table 4:** Compressive strength results.

Concrete Mix		Compressive Strength, *f*_c_ (N/mm^2^)	*f*_cm_ (N/mm^2^)	Relative Range (%)
Control Cracking Tests (*V_f_* = 0.11%)	PPF-CC	40.2	40.0	1.5%
		40.1		
		39.6		
	RSF-CC	39.9	39.7	8.6%
		37.9		
		41.3		
Residual Strength Tests (*V_f_* = 0.26%)	SF-RS	33.1	34.5	6.4%
		35.0		
		35.3		
	RSF-RS	35.5	35.3	1.1%
		35.4		
		35.1		

**Table 5:** Potential for cracking of the studied fiber reinforced mixes.

Concrete Mix	Average Age of First Crack after Drying, *t*_cr_ (Days)	Average Initial Strain (×10^−6^)	Average Maximum Strain (×10^−6^)	Average Stress Rate, S (MPa/day)	Potential for Cracking by Age of First Crack	Potential for Cracking by Average Stress
PPF-CC	30	−4	−84	0.13	Low	Moderate Low
RSF-CC	34	−6	−89	0.13	Low	Moderate Low

**Table 6:** Flexural residual strength according to EN 14651 [58].

Concrete Mix	Max. Load, *F*_L_ (kN)	Load at CMOD = 0.5 mm, *F*_1_ (kN)	Load at CMOD = 2.5 mm, *F*_1_ (kN)	LOP (N/mm^2^)	*f*_R,1_ (N/mm^2^)	*f*_R,3_ (N/mm^2^)
SF-RS	10.6	4.6	2.3	3.6	1.5	0.8
	11.0	3.7	1.8	3.6	1.2	0.6
	10.3	4.3	2.3	3.5	1.5	0.8
Mean				3.6	1.4	0.7
Relative Range				2.8%	21.4%	28.6%
RSF-RS	12.1	4.3	2.1	4.1	1.4	0.7
	11.3	4.2	2.2	3.8	1.4	0.8
	12.3	4.8	2.2	4.3	1.7	0.8
Mean				4.1	1.5	0.8
Relative Range				12.2%	20.0%	12.5%

## Data Availability

Data available on request due to privacy restrictions. The data presented in this study are available on request from the corresponding author. The data are not publicly available since its use is subjected to approval by the contract between the funding entity and CSIC.
